# Ala^®^sil chemical characterization and toxicity evaluation: an example of the need for the Medical Device Regulation 2017/745

**DOI:** 10.3389/fphar.2023.1310463

**Published:** 2024-01-11

**Authors:** Cristina Andrés-Iglesias, Ivan Fernandez-Bueno, Salvador Pastor-Idoate, Rosa M. Coco-Martin, J. Carlos Pastor

**Affiliations:** ^1^ Instituto Universitario de Oftalmobiología Aplicada (IOBA), Retina Group, Facultad de Medicina, Universidad de Valladolid, Valladolid, Spain; ^2^ Centro en Red de Medicina Regenerativa y Terapia Celular de Castilla y León, Valladolid, Spain; ^3^ Red de Investigación Cooperativa Orientada a Resultados en Salud (RICORS), Red de Enfermedades Inflamatorias (REI), Instituto de Salud Carlos III, Madrid, Spain; ^4^ Department of Ophthalmology, Hospital Clínico Universitario de Valladolid, Valladolid, Spain

**Keywords:** ophthalmic medical devices, low-molecular-weight components, toxicity, silicone oil, polydimethylsiloxane, MDR

## Abstract

**Introduction:** Ala^®^sil infusion was on the market for clinical use under the Medical Devices Directive (MDD) 93/42/EEC as an irrigating solution based on polydimethylsiloxane (PDMS). The product was withdrawn in 2016, and to the best of our knowledge, it did not cause any health damage.

**Methods:** A bibliographic review and experimental analysis were conducted to evaluate whether this CE-marked product could have been used in patients under the current Medical Device Regulation (MDR) 2017/745. Analytical results from gas chromatography–mass spectrometry (GC-MS) and matrixassisted laser desorption ionization (MALDI) were performed. Citotoxicity studies were also carried out.

**Results:** Only one study related to Ala^®^sil clinical use was found, describing a pilot series of five patients. The authors rated the product as not helpful in three out of the five cases for internal searching of retinal breaks and in four out of the five cases for drainage of subretinal fluid. No other scientific papers or documentation was found regarding Ala^®^sil’s safety. Nevertheless, the product was introduced in the market after achieving the CE marking. GC-MS and MALDI showed that the polymer has a low molecular weight of 1,000 g/mol. Several linear and cyclic low-molecular-weight components (LMWCs) were identified as impurities ranging from L3 to D8, with a molecular weight below 600 g/mol. The Ala^®^sil sample was found to be cytotoxic after 24 h of cell culture but non-cytotoxic after 72 h, probably due to the cellular regeneration capacity of an immortalized cell line. Tissular cytotoxicity revealed an increased apoptosis rate but without morphological modifications.

**Discussion:** Although Ala^®^sil cannot be classified as cytotoxic, this substance appears to increase retinal cell death processes. This study supports the notion that the MDDwas not functioning adequately to ensure the safety of medical devices. However, the current MDR 2017/745 imposes stricter standards to prevent the commercialization of medical devices without high-quality preclinical and clinical information, as well as precise clinical verification for their use, information not available for Ala^®^sil infusion.

## 1 Introduction

Ala^®^sil infusion, according to its instructions for use (IFU), was an irrigation solution based on polydimethylsiloxane (PDMS) (Alamedics, 2023). PDMS is commonly used in medical devices as an infusion liquid because of its biocompatibility, chemical stability, and low toxicity (Miranda et al., 2022). As an infusion fluid, PDMS can be used as a drug delivery vehicle and a blood-contacting biomaterial (Subramaniam and Sethuraman, 2014). In ophthalmology, PDMS is used as a material in medical devices such as intraocular lenses, contact lenses, and vitreous substitutes (Lin et al., 2014; Jellali et al., 2017; Chen et al., 2021). As silicone oil, PDMS has been used as a long-term tamponade to treat complicated vitreoretinal diseases (Januschowski et al., 2018; Chen et al., 2021). However, PDMS chains with molecular weight <1 kDa (low-molecular-weight components) are synthesized during the manufacturing process (Mendichi et al., 2019) and can increase emulsification and, if diffused into ocular tissues, create inflammatory reactions (Nakamura et al., 1991; Pastor et al., 1998; Mendichi et al., 2019; Dresp, 2021; Steel et al., 2021).

During the time of Ala^®^sil commercialization, the Medical Devices Directive (MDD) 93/42/EEC was in force. However, its application revealed several formal defects not only in the ophthalmic products but also in others (Januschowski et al., 2018; The International Consortium of Investigative Journalists, 2023). In ophthalmology, toxic batches of perfluorocarbon liquids (PFCLs) caused hundreds of blind cases worldwide, causing a real concern among retinologists (Méndez-Martínez et al., 2018; Tobalem et al., 2020). Toxicity was not revealed by the cytotoxicity tests used, although it was accomplished with the ISO guidelines (Srivastava et al., 2018; Menz et al., 2019), and new methodologies for cytotoxic and chemical evaluation have had to be developed, allowing the specific identification of the impurities causing these toxic effects (Srivastava et al., 2020; Coco-Martin et al., 2021). In this context, the value of biological tests is emphasized, employing cells in direct contact. This approach is deemed significant because this particular medical device directly engages with the retina during its clinical application (Ruzza et al., 2019; Romano et al., 2021). Other authors have suggested that healthcare providers should require purity tests from companies to better select the products to use for their patients (Steel et al., 2021). However, the confidence of doctors and surgeons must rest on what the CE marking represents, which is an authentic guarantee of quality and safety. This marking must be a sign of trust between the patients and healthcare agents.

In this context, since May 2021, all manufacturers of medical devices who wish to obtain the CE marking have to follow the rules of the new Medical Device Regulation (MDR) 2017/745 [Regulation (EU) 2017/745 (EU MDR), n. d.]. The MDR is intended to avoid, among other objectives, safety concerns such as the acute toxicity caused by some perfluorocarbon liquids (Pastor et al., 2017; Coco et al., 2019) or toxicity caused by some inner limiting membrane stains (Gerding, 2016). Hence, the MDR emphasizes the necessity for manufacturers to guarantee that their medical devices possess a justification for the intended use. This information should be substantiated by pertinent preclinical and clinical data or other studies, reports, and clinically relevant information found in the scientific literature of a device, for which equivalence to the given device can be demonstrated [Regulation (EU) 2017/745 (EU MDR), n. d.].

In this regard, the transition from the MDD to the MDR represents a substantial regulatory overhaul, evident in the increased scope and depth of the MDR. The MDR focuses on the entire product lifecycle, encompassing the development, testing, manufacturing, commercialization, efficacy, safety, and long-term use. The prominence of the term “safety” is markedly elevated in the MDR, appearing 290 times compared with the MDD’s 40 occurrences. These changes significantly impact companies in the medical device industry, requiring them to reassess their portfolios and conduct a global impact assessment for compliance. Annex I of the MDR specifies new safety and performance requirements, necessitating the re-certification of existing devices, particularly those previously CE-marked under the MDD. Manufacturers face the challenge of generating more in-depth clinical and preclinical data to meet the heightened safety and performance standards. The reporting landscape is undergoing a transformation, requiring incidents, injuries, and deaths to be reported to the centralized EU portal, EUDAMED, with revised timelines. This study assesses whether Ala^®^sil could have been brought to the market for clinical application in accordance with the current MDR, examining both its safety and potential effectiveness. Through a bibliographic review, this study aims to define its clinical use, design characteristics, and chemical and cytotoxic characterization using cellular and tissue models according to ISO standards.

## 2 Materials and methods

### 2.1 Sample information

The Ala^®^sil (Alemedic, Dornstadt, Germany) sample arrived in its original box containing a sealed 70-mL bottle of the product to be tested. This product corresponds to polydimethylsiloxane (PDMS), lot number INF 220413, with the expiration date of 04/30/2016, EAN: 04250736800801, and code AMSILINFUSIO, and, according to the packaging, it was distributed in Spain by Bloss S.A. (Barcelona, Spain). It came with its corresponding IFU (Alamedics, 2023).

### 2.2 Literature review

The literature review searched for preclinical and clinical studies performed on Ala^®^sil and was developed in the PubMed, Science Direct, Scopus, and ClinicalTrials.gov electronic databases from January 1990 to December 2022. Potentially relevant articles were sought using the following search terms in combination: medical subject headings terms and text words: silicon oil infusion, silicon oil irrigation, continuous silicon infusion, low viscosity silicon oil, preoperative tool + ophthalmology, and Ala^®^sil. No language restrictions were applied. We also scanned the reference lists of the retrieved publications to identify additional relevant articles (cross-reference strategy), used the MEDLINE option “Related Articles,” and consulted review articles on the topic to supplement the search.

### 2.3 Sample analysis by headspace gas chromatography–mass spectrometry (HS-GC-MS)

For the HS-GC-MS analysis of Ala^®^sil, 2 mL of the sample was added to 20-mL dark vials sealed with silicone septa (Merck, United States of America). The samples were analyzed in triplicate. LMWC analysis was performed following the Agilent Technologies application note for oligosiloxanes in silicon oil (Agilent-Technologies, 2011). The headspace incubation time was set at 110 °C for 60 min, and the syringe temperature was 115 °C. Then, 0.5 mL of the sample was injected using the coupled HS autosampler (CombiPAL, CTC) into a 7890B GC system gas chromatograph (Agilent Technologies, United States of America), and the inlet temperature was set at 250°C, connected to a 5977A MSD (Agilent Technologies, United States of America) single quadrupole mass spectrometer. For gas chromatographic separation, an HP-5ms capillary column of 30 m × 0.25 mm × 0.25 μm (Agilent Technologies, United States of America) was used; the chromatography oven was set as follows: initial temperature 50°C during 5 min and then raised to 160°C at 10°C/min, raised to 220°C at 20°C/min, and maintained during 20 min. Detection and data acquisition were performed in the scan mode from 40 to 600 Da, with MS source 230°C and MS quad 150°C. Data analysis was performed using MassHunter Data Acquisition software (quantitative analysis B.07.00, Agilent Technologies, United States of America). The impurities were identified using the NIST17 MS search 2.3 library and the EIC of the characteristic ions. Identification of the L2, D3, D4, and D5 compounds was performed by comparison with the standards (Sigma-Aldrich, United States of America). Standard calibration curves were used to quantify L2 (*R* ^2^ = 0.9969), D3 (*R* ^2^ = 0.9967), D4 (*R* ^2^ = 0.999), and D5 (*R* ^2^ = 0.9947) (calibration curves can be found in Supplementary Material S1). For the other linear and cyclic oligosiloxanes (L3, L4, L5, L6, L7, D6, D7, and D8), a semi-quantitative approach was taken using the L2 calibration curve for the linear oligosiloxanes L3, L4, L5, and L6; and the D5 calibration curve for the cyclic oligosiloxanes D6, D7, and D8.

### 2.4 Sample analysis by matrix-assisted laser desorption ionization–time of flight detector (MALDI-TOF)

Mass spectra were acquired on an Autoflex Speed mass spectrometer (Bruker Daltonics, Bremen, Germany) using a Smartbeam^TM^ laser as the ionization source. The acceleration voltage was 20 kV in the reflection mode. We accumulated 2,000 shots in the positive mode for all spectra in the range of m/z 200–2000 Da. All the samples were analyzed using 2,5-dihydroxybenzoic acid (2,5-DHB) as the matrix. Data analysis was performed using flexAnalysis 3.4 and PolyTools 1.0.

### 2.5 Cytotoxicity evaluation

#### 2.5.1 Human retinal pigment epithelial cells (ARPE-19 cells)

Direct contact cytotoxicity tests were performed as previously described by our group (Srivastava et al., 2018; 2020; Coco et al., 2019). In brief, cultures of the human retinal pigment epithelial cell line (ARPE-19) were prepared in 96-well culture plates, followed by 24-h cell cycle synchronization in a fetal bovine serum (FBS)-free cell culture medium (Gibco, UK). The cultures were then exposed directly to the samples for 30 min. After exposure, the samples and the culture medium were removed from each well and washed to remove any remnants. Then, the cells were incubated for 24 h and 72 h for cell growth. Subsequently, viability was measured by the MTT assay, as previously described (Srivastava et al., 2018).

All the experiments were performed following the ISO guidelines and under Good Laboratory Practices certification. Viability values <70% were considered cytotoxic, according to UNE-EN ISO 10993-5. Data were analyzed by calculating each well’s optical density value of cell culture viability, which was recorded with a SpectraMax M5 Microplate Reader (Molecular Devices, United States of America).

#### 2.5.2 Porcine neuroretina explants

Fresh eyes (n = 3) were obtained from pigs aged 6–8 months from a local slaughterhouse. Neuroretina (NR) explants were obtained, as described (Di Lauro et al., 2016), and directly exposed to the test samples (Ala^®^sil) or phenol (positive control; Sigma-Aldrich) for 30 min. Then, the NR explants were laid on Transwell membranes 24 mm in diameter with a 0.4-mm-pore polycarbonate membrane insert (Corning Life Sciences) with the photoreceptor layer facing the membrane and maintained at culture conditions until 72 h, as previously described (Usategui-Martín et al., 2020). In parallel, NR explants were extracted and processed (fresh NR) or cultured without exposure to any substance (not exposed) as the negative controls. Experiments were run in triplicate; three retinal explants were used for each experimental condition; therefore, 12 retinal explants were used.

NR explants were fixed with 4% paraformaldehyde (PANREAC Química S.L.U., Barcelona, Spain) for 2 h at 4 °C and then embedded in paraffin (Paraplast Plus, Leica Biosystems, Nussloch, Germany) using an automatic tissue processor (ASP300; Leica Microsystems, Wetzlar, Germany). Paraffin-embedded sections were deparaffinized in xylene (Sigma-Aldrich) and rehydrated in decreasing ethanol concentrations. The sections were then stained with hematoxylin and eosin (Sigma-Aldrich). As previously described, the tissue and cellular morphological modifications of the neuroretina explants were qualitatively graded according to UNE-EN ISO 10993-5 (Pastor et al., 2017). A single masked researcher performed this analysis, and scores more outstanding than two suggested a cytotoxic effect according to the UNE-EN ISO guidelines.

Furthermore, to quantify cellular death, TUNEL immunostaining was performed with an *in situ* cell death detection kit (Roche Diagnostics, Mannheim, Germany). Nuclei were counterstained with DAPI. TUNEL analysis was performed in triplicate with four samples/for each experimental condition. Control slides in which primary antibodies were omitted were also processed in parallel. To quantify the TUNEL assay, immunofluorescence micrographs were acquired at the same exposure, intensity, and gain (magnification × 40 images: n = 8 sections per experimental condition). Then, the TUNEL-stained nuclei were manually counted in each nuclear layer using ImageJ software (1.49 version; National Institutes of Health). Finally, the TUNEL-labeled nuclei were correlated with the total DAPI-stained nuclei to obtain quantifiable percentage results. A single masked researcher performed the analysis.

### 2.6 Data acquisition and analysis

All data were collected in an Excel database (Microsoft Office Excel, 2016; Microsoft Corporation, Redmond, WA, United States of America). SPSS (version 24.0, SPSS Inc., Chicago, IL, United States of America) was used for statistical analyses. After confirming the homogeneity of variance and normal distribution of the data, ANOVA, followed by pairwise comparisons, was carried out (Tukey test). Differences were considered statistically significant at *p* < 0.05. Data are expressed as means ± SEM.

## 3 Results

After an extensive literature review, we did not find any references to support the specific information in the product IFU (Alamedics, 2023). To the best of our knowledge, there is only one paper, with a short series of cases (five patients) (Wong et al., 2011). However, the authors also recognize the inherent risk of using this product (Wong et al., 2011). The adverse effects reported include increased intraocular pressure due to a steroid response, Ala^®^sil viscosity reduction at body temperature (from 5 to 0.8 mPa), and intraocular pressure drop during silicone oil infusion at vitreous fluid aspiration. In addition, they reported that if traces of Ala^®^sil remain, it can mix with the conventional silicone oil and promote emulsification; therefore, it must be removed entirely. Finally, the authors did not select cases with retinal breaks to avoid the infusion of the low-molecular-silicone oil under the retina (Wong et al., 2011). There are no references to the chemical analysis of the substance in this paper, and no document has been found to prove its equivalence. This unique pilot series of five patients was published by a single surgeon who declared interest in a patent on the product.

According to the IFU (Alamedics, 2023), Ala^®^sil infusion is a colorless, homogeneous liquid composed of ultrapure PDMS with the formula CH3 [Si(CH_3_)_2_O]n-Si(CH_3_)3, where n is the number of monomer units [SiO(CH_3_)2]. Ala^®^sil infusion is chemically and physiologically inert and has a density of 0.9 g/cm^3^ and a viscosity of 5 mPas, which corresponds to 5 cst.

**FIGURE 1:**
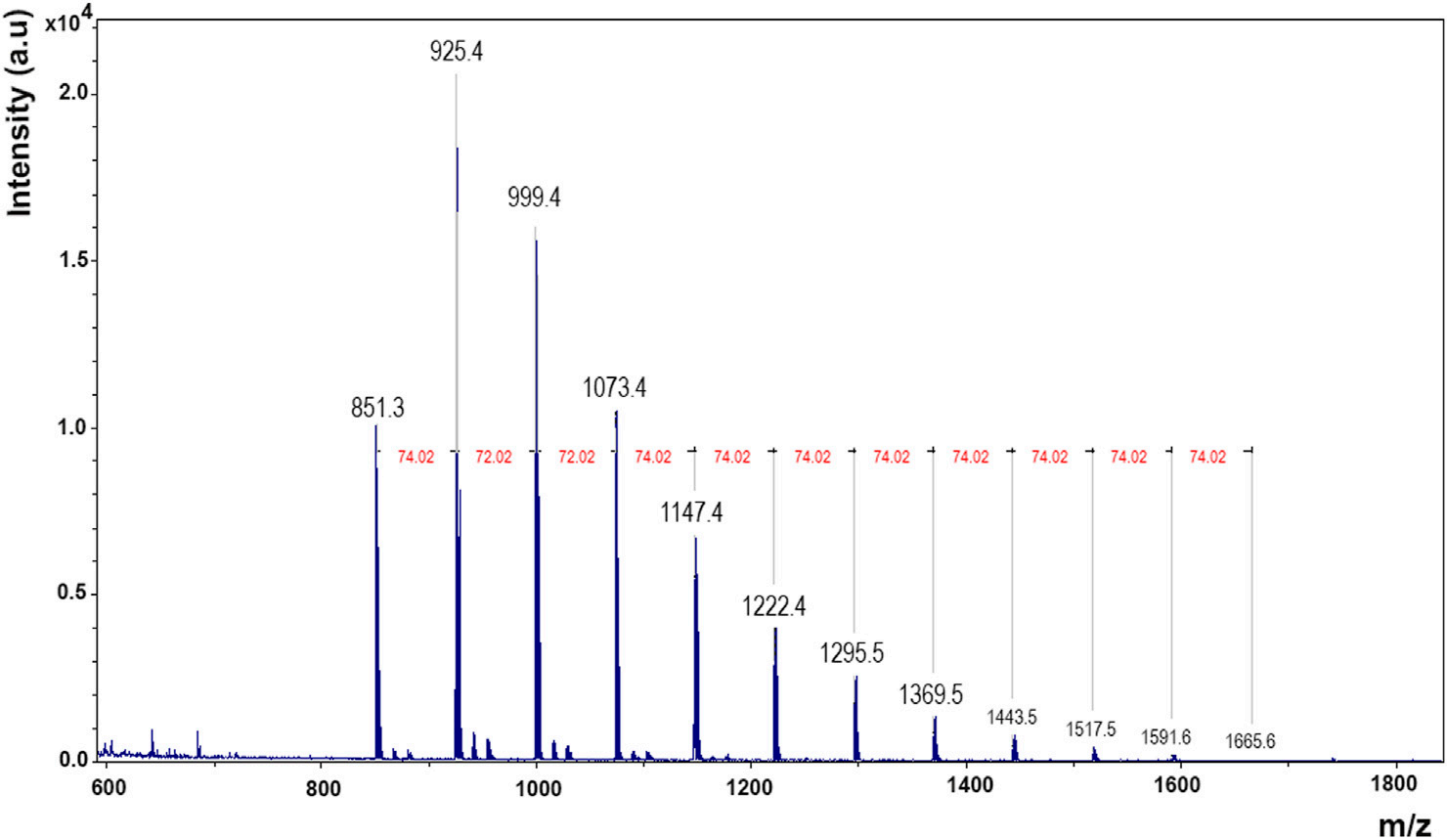
MALDI spectrum of the polymer. Mass charge (m/z) vs. intensity. Gaussian bell characteristic of polymers and siloxane repeating units (74.02 m/z).

To analyze the polymer characteristics, the MALDI-TOF analysis was performed. The sample spectrum showed a PDMS polymer with repetitive units of C_2_H_6_OSi (74 m/z) (Figure 1). Data analysis of the sample showed that the most abundant distribution corresponded to a number average molecular weight (Mn) of 992.23 g/mol, a weight average molecular weight (Mw) of 1005.10 g/mol, and a polydispersity index of 1.01.

**FIGURE 2:**
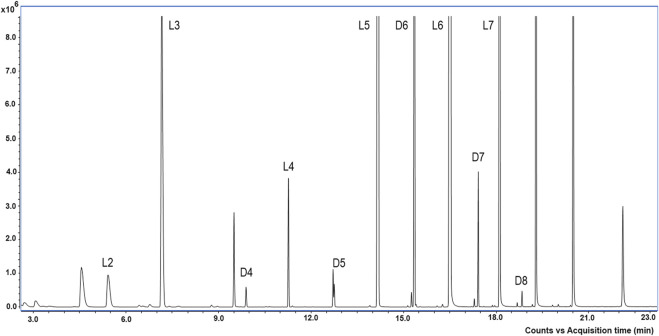
TIC scan chromatogram of the Ala^®^sil sample. Counts (8.6 × 10^6^) vs. acquisition time (minutes). Identified LMWCs were D3: hexamethylcyclotrisiloxane, L3: octamethylcyclotrisiloxane, D4: octamethylcyclotetrasiloxane, L4: decamethyltetrasiloxane, D5: decamethylcyclopentasiloxane, L5: dodecamethylpentasiloxane, D6: dodecamethylcyclohexasiloxane, L6: tetradecamethylcyclooctasiloxane, D7: tetradecamethylcycloheptasiloxane, L7: hexadecylcycloheptasiloxane, D8: hexademethylcyclooctasiloxane, and L8: hexadecamethyloctasiloxane.

To evaluate the cyclic and linear oligosiloxanes, the HS-GC-MS analysis was performed. The chromatogram showed that the Ala^®^sil sample contained many volatile LMWCs (Figure 2). In the analyzed sample, several linear (from L3 to L7) and cyclic (from D3 to D8) oligosiloxanes with Mw below 600 g/mol were found (Table 1). The concentration of the identified linear and cyclic LMWC (L3 to D8) in the Ala^®^sil sample was 5308.84 ppm ± 7.06 ppm (6% error).

**TABLE 1:** Linear and cyclic low-molecular-weight compounds (LMWCs) identified in Ala^®^Sil by HS-GC-MS. Names of the LMWC, retention time (Rt), molecular formula, molecular weight (Mw), and spectrum match values form NIST mass spectra library Match and Reverse Match (R.Match).

LMWC	Name	Rt	Formula	Mw	Match	R.Match
D3	Hexamethylcyclotrisiloxane	5.413	C_6_H_18_O_3_Si_3_	222	935	938
L3	Octamethyltrisiloxane	7.161	C_8_H_24_O_2_Si_3_	236	916	916
D4	Octamethylcyclotetrasiloxane	9.888	C_8_H_24_O_4_Si_4_	296	928	945
L4	Decamethyltetrasiloxane	11.265	C_10_H_30_O_3_Si_4_	310	948	948
D5	Decamethylcyclopentasiloxane	12.753	C_10_H_30_O_5_Si_5_	370	911	917
L5	Dodecamethylpentasiloxane	14.172	C_12_H_36_O_4_Si_5_	384	909	910
D6	Dodecamethylcyclohexasiloxane	15.353	C_12_H_36_O_6_Si_6_	444	941	988
L6	Tetradecamethylhexasiloxane	16.507	C_14_H_42_O_5_Si_6_	458	868	919
D7	Tetradecamethylcycloheptasiloxane	17.423	C_14_H_42_O_7_Si_7_	518	808	823
L7	Hexadecamethylheptasiloxane	18.106	C_16_H_48_O_6_Si_7_	532	861	909
D8	Hexadecamethylcyclooctasiloxane	18.842	C_16_H_48_O_8_Si_8_	592	857	875
L8	Hexadecamethyloctasiloxane	19.241	C_16_H_50_O_7_Si_8_	578	808	725

A comparison was conducted between m/z profiles obtained by MALDI and Mw of the LMWC found in GC-MS. It has to be considered that MALDI-TOF operating in a positive mode with DHB as the matrix allowed the acquisition of positive-ion mass profiles. In contrast, GC-MS facilitates compound identification by their molecular weight and the m/z values in the chromatogram spectra. The comparison process involved normalizing MALDI-TOF data, subtracting the matrix peaks, and considered the potential addition of ions to align with GC-MS Mw data (Supplementary Material, Supplementary Tables S2.1, S2.2). Thus, the predominant ionization species commonly observed in the positive ion mode is the protonated ion [M + H]^+^, and other forms are possible as the DHB matrix can form complexes with sodium cations, leading to the formation of sodium ions [M + Na]^+^. Although not common, the [M] value could be observed at low ranges of the spectra. The MALDI-TOF spectra for lower ranges can be observed in Supplementary Material slx.

**FIGURE 3:**
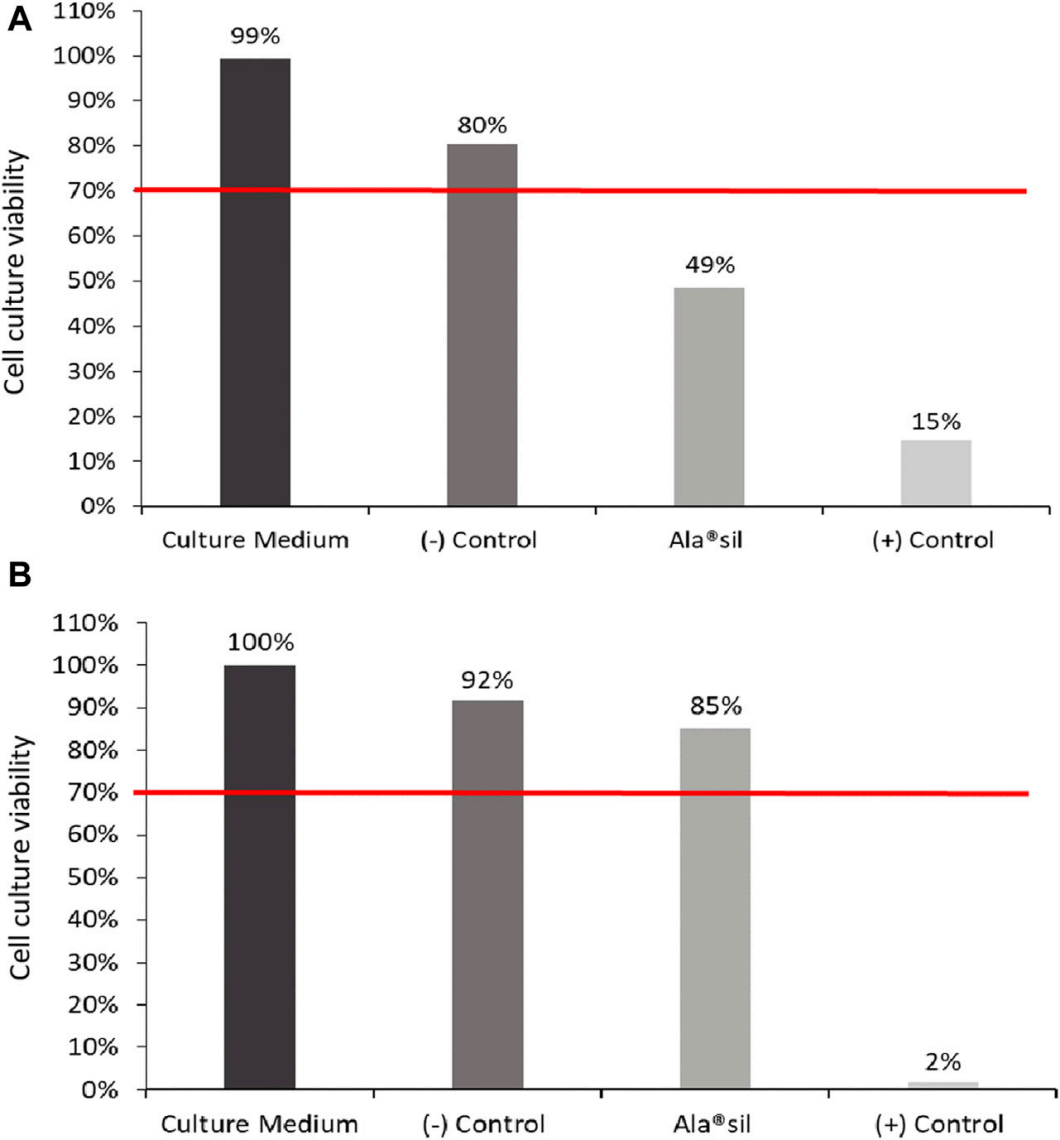
Cell culture viability assessed by the MTT assay. Cell cultures were exposed to the samples for 30 min, followed by 24 **(A)** and 72 **(B)** hours of incubation for cell growth.

**FIGURE 4:**
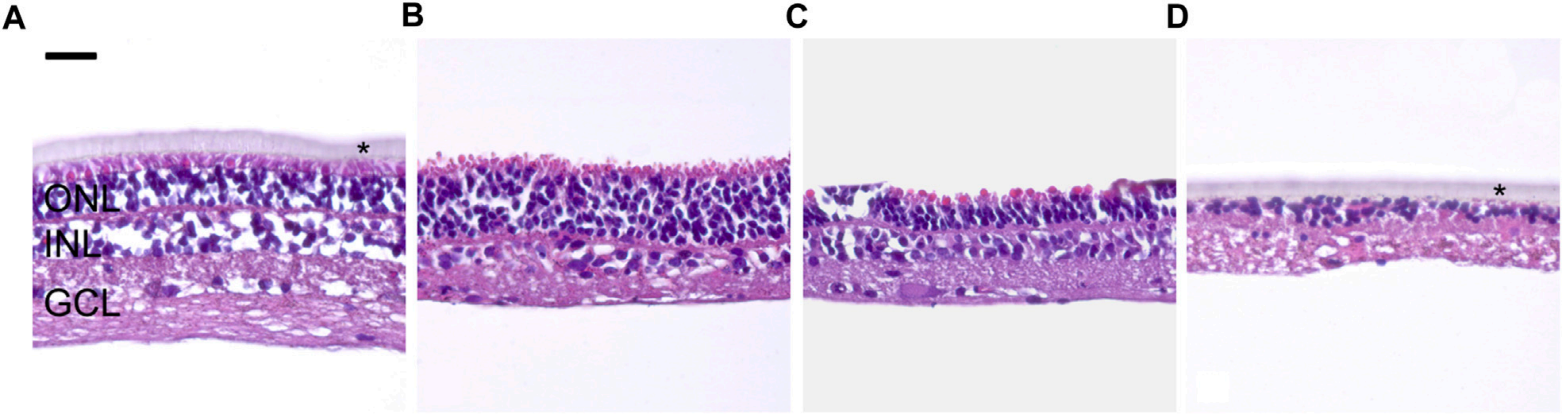
Morphological modifications of neuroretina explants. **(A)** Before culture, fresh NR structure and general morphology were adequately preserved (grade 0–1). **(B)** Not-exposed NR showed a loss of photoreceptor outer segment and partial photoreceptor inner segment edema with an incipient reduction in the number of nuclei at the INL and beginning of retinal tissue vacuolization (grade 1–2). **(C)** NR exposed to Ala^®^sil and cultured for 72 h showed similar morphological modifications as the not-exposed NR **(B)**. **(D)** NR exposed to phenol showed a significant loss of retinal structure, cell nuclei, and retinal parenchyma (grade 4). GCL: ganglion cell layer; INL: inner nuclear layer; ONL: outer nuclear layer; scale bar: 25 μm *Transwell membranes.

It should be noted that GC-MS and MALDI-TOF are analytical techniques with different principles and use two completely different ionization methods. This disparity makes direct comparisons difficult, especially at low molecular weights, due to the presence of the matrix in MALDI-TOF. GC-MS involves the separation of volatile compounds by chromatography, followed by electron impact ionization. In contrast, MALDI-TOF is optimized for larger, less volatile molecules using laser-induced desorption and matrix-assisted ionization. Variations in sample preparation, ionization mechanisms, matrix effects, mass range, sensitivity, and resolution make it even more difficult to compare the results between the two techniques. Consequently, only compounds D3 with [M + H]+ and [M + Na]+ and perhaps L3 and L5 with [M] were tentatively identified in MALDI (Supplementary Material S2.2). Regarding the biological evaluation of the product, the cell culture analysis after 30 min of direct contact with Ala^®^sil and 24 h of posterior cell growth showed that the Ala^®^sil sample was cytotoxic with a viability value of 49% (Figure 4A). However, after 30 min of direct contact and 72 h of posterior cell growth, the Ala^®^sil sample was not found to be cytotoxic, showing a viability value of 85% (Figure 3A).

Neuroretina evaluation of the fresh porcine samples (Figure 4A) had a clearly defined, layered retinal structure and adequate cellular preservation before culture, corresponding to a degeneration degree of 0–1. The structure of the NRs that were not exposed (Figure 4B), and after direct contact with Ala^®^sil (Figure 4C), revealed a loss of photoreceptor outer segment and partial photoreceptor inner segment edema, reduction in the number of nuclei in the inner nuclear layer, and the beginning of retinal tissue vacuolization. These modifications corresponded to 1–2 degeneration degrees, according to ISO 16672 (ISO, 2020). NR, after direct contact with phenol (Figure 4D), showed a significant loss of retinal structure, loss of cell nuclei, and loss of retinal parenchyma, corresponding to a degeneration degree of 4.

**FIGURE 5:**
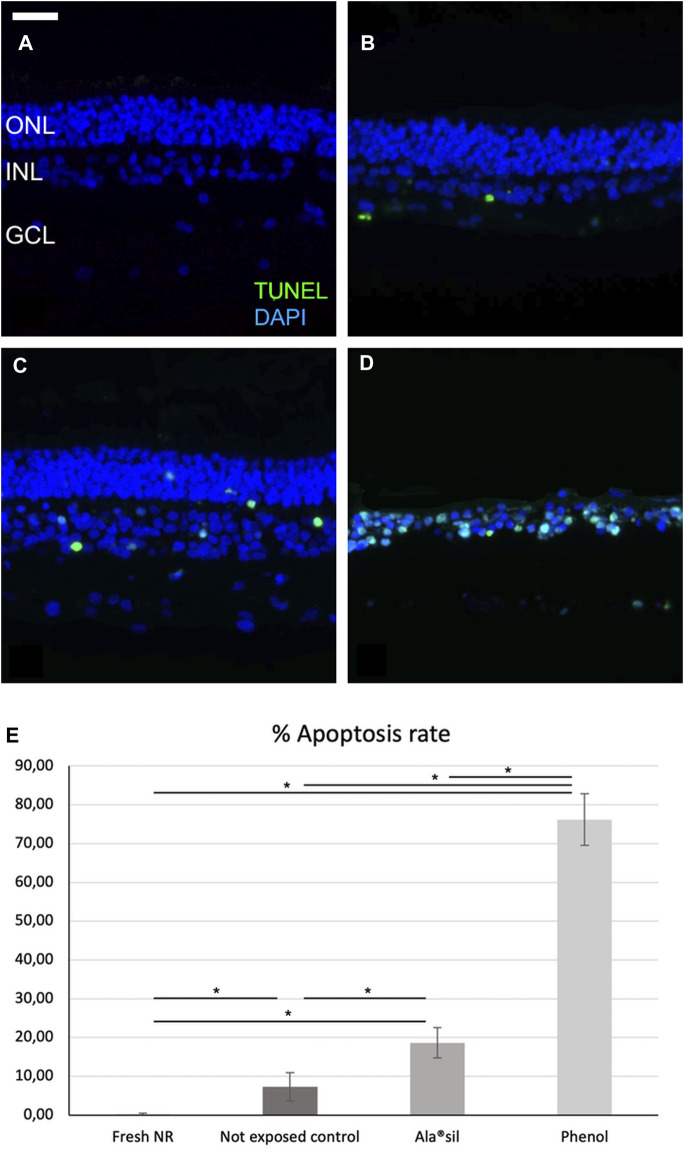
Apoptosis characterization in neuroretina explants. **(A)** Fresh NR did not show TUNEL immunoreactivity. **(B)** Not-exposed NR showed detectable labeling at the GCL and the INL. **(C)** In NR exposed to Ala^®^sil or **(D)** phenol (Fig. 6D), TUNEL labeling was observed in all the retinal layers. **(E)** Apoptosis rate in the total retinal layers was significantly higher in NR exposed to phenol or Ala^®^sil compared to fresh NR and those not exposed. *p < 0.05. GCL: ganglion cell layer; INL: inner nuclear layer; ONL: outer nuclear layer; scale bar: 25 μm.

The apoptosis analysis performed with the TUNEL detection kit labels DNA strand breaks in cells that undergo apoptosis (Gavrieli et al., 1992). TUNEL immunoreactivity was undetectable in fresh NR (Figure 5A). In the case of not exposed NR, TUNEL immunoreactivity was noticeable at the GCL and INL (Figure 5B), while in NR exposed to Ala^®^sil (Figure 5C) or to phenol (Figure 5D), labeling was detected in all the retinal layers. Data from the TUNEL analysis were evaluated, obtaining apoptosis rate results in the total retinal layers significantly higher (*p* < 0.05) in NR exposed to phenol with results of 76.19% apoptosis rate of total retina ± 6.65% standard deviation and Ala^®^sil with an 18.63% apoptosis rate of total retina ± 3.92% standard deviation compared to fresh NR (0.23% apoptosis rate of total retina ± 0.22% standard deviation) and those not exposed (7.31% apoptosis rate of total retina ± 3.61% standard deviation).

## 4 Discussion

Ala^®^sil infusion was introduced into the healthcare market for its application as an infusion liquid during vitrectomies, aiming to prevent intraocular hemorrhages and manage challenging cases of ocular trauma. However, the available information about this product was limited.

The MDR presents stricter criteria and clearer guidelines for medical device classification mainly based on the risk, and some devices have been reclassified (Annex VIII). In the case of Ala^®^Sil infusion, the classification should be a surgically invasive device for transient use (short term), and it will be classified as Class IIa, requiring a notified body approval and conformity assessment of the technical documentation. In this regard, manufacturers need to demonstrate that their medical device meets the requirements by conformity assessment based on a quality management system and an assessment of technical documentation (Annex IX), type examination (Annex X), and/or product conformity validation (Annex XI). Here are the key considerations for the documentation of Class IIa devices compared to the previous MDD:

a. Clinical evidence: manufacturers should provide clinical evidence to demonstrate the safety and performance of their devices. Manufacturers are required to provide comprehensive clinical data, including detailed clinical evaluations, post-market clinical follow-up plans, and systematic literature reviews, which assess the relevant scientific literature and clinical data pertaining to the device’s intended use. The MDR places increased emphasis on the clinical evaluation process compared with the MDD, ensuring a thorough and systematic assessment of the available clinical data.
b. Technical documentation: the technical documentation for Class IIa devices should contain detailed information about the device’s design, intended purpose, risk management, and essential performance characteristics. The technical documentation must also include a biological evaluation, which involves assessing the biological safety of the medical device.

As explained above, sufficient clinical evidence of the usefulness and safety of the medical device is necessary. Therefore, we conducted an extensive literature search for clinical evidence. However, as mentioned, we found only one scientific manuscript related to Ala^®^sil (Wong et al., 2011). This clinical paper does not adequately verify the necessity of this product by claiming advantages that were not justified in that paper. The product was tested for break localization, vitreous base dissection, and drainage of subretinal fluid in only five patients. Three patients exhibited long-standing retinal detachment, another patient presented with diabetic retinal detachment along with rhegmatogenous retinal detachment, and the final case involved a giant retinal break. The surgeon evaluated that the product contributed to the most thorough removal of the vitreous base in all five cases. However, the product was useless in three of the five cases for internal search of retinal break application and four over five cases for drainage of subretinal fluid (Wong et al., 2011). This seems to be insufficient clinical support. Furthermore, the indications claimed on the commercial product are not supported by that paper. Fortunately, the follow-up of these five patients did not show adverse events related to its use.

Due to the lack of the scientific literature that would support a clear clinical evidence of its intended use in the market and its safety, it was decided to conduct a study on the characteristics of the material (chemical composition) and its biological safety. In this regard, we analyzed the medical device to provide a detailed composition, and we also conducted biocompatibility tests of this CE-marked medical device.

Because it is a medical device based on the PDMS polymer, the potential toxicity of LMWCs must be considered. It has been a well-established issue since the early 1990s (Nakamura et al., 1991; Pastor et al., 1992; 1998; Mendichi et al., 2019). Its capacity to diffuse into the surrounding tissues has been reported (Nakamura et al., 1991), and it is assumed that they are related to the rate of emulsification and intraocular inflammatory reactions (Mendichi et al., 2019). Another possible harmful effect of LMWCs could be related to their ability to facilitate the denaturation and aggregation of human serum albumin and, presumably, other human blood proteins present in the vitreous cavity during surgery (Romano et al., 2020). In the case of silicone oil (PDMS with a high degree of polymerization), polymer chains with molecular weight <10,000 g/mol (LMWC), and particularly cyclic or linear LMWC with a molecular weight <1,000 g/mol, are considered “impurities” (Mendichi et al., 2019). In this regard, the molecular weight of Ala^®^sil was 1,005 g/mol, which would be within the LMWC category, while also having a high concentration of linear and cyclic LMWC below 600 g/mol, as demonstrated by MALDI and HS-GC-MS.

Moreover, volatile components such as short-chain cyclic oligosiloxanes D4, D5, and D6 have been recognized as toxic compounds by the European Chemical Agency (ECHA) and have been listed in the Candidate List of Substances of Very High Concern for Authorization (Mendichi et al., 2019; Romano et al., 2020; Dresp, 2021; European Chemicals Agency, 2023). These compounds (D4, D5, and D6) are part of the composition of the Ala^®^sil PDMS sample. In addition, silicon oil has been reported to be non-cytotoxic at a concentration of 1,493.75 ppm LMWC (Romano et al., 2020). However, Ala^®^sil is a polymer with a high rate of linear and cyclic oligosiloxanes with Mw < 600 and LMWC values of 5,319.5 ppm ± 7.08 (6% error) (L3 to D8). This corresponds to a concentration 3.5 times higher than the LMWC concentration tested by Romano et al. (2020).

Regarding the biological assays, cell cytotoxicity tests showed that Ala^®^sil was cytotoxic after 24 h of cell growth. ISO guidelines consider a sample cytotoxic with viability values below 70% (ISO, 2009). However, after 72 h of cell growth, the sample showed non-cytotoxicity, probably due to the cellular regeneration capacity of the ARPE-19 immortalized cell line after the next 48 h of growth, a fact that has already been pointed out by our group (Srivastava et al., 2018; Coco et al., 2019). The neuroretina spontaneously degenerates during culture (Fernandez-Bueno et al., 2008), so the morphological changes observed in the neuroretinas exposed to Ala^®^sil were similar to that in the not-exposed control. However, specific evaluation and quantification of apoptosis revealed significantly higher levels in Ala^®^sil-exposed NR. Therefore, although Ala^®^sil cannot be classified as cytotoxic (Pastor et al., 2017), according to UNE-EN ISO 10993-5:2009, this substance seems to increase the processes of retinal cell death. In this regard, we hypothesize that if we extend the culture time of the samples to reveal cell death, morphological changes will be observed, and Ala^®^Sil can be classified as cytotoxic.

Therefore, it is evident that Ala^®^sil presented a series of weaknesses that should have required a detailed justification before being approved for the CE mark. This product is an element of a series of medical devices manufactured by AlaMedics. Some cases of suspected toxicity have been reported with a dye for internal limiting membranes (AlaPurple^®^), which was also marketed by the same company. It seemed that the concentration of the product finally dealt with was higher than that which had been experimentally tested (Green, 2015; Ärzteblatt, 2015; Gerding, 2016; Januschowski et al., 2018). In addition, this company manufactured the perfluorocarbon liquid AlaOcta^®^; some batches of this product were highly cytotoxic, leaving hundreds of people blind worldwide (Pastor et al., 2017; Coco et al., 2019; Srivastava et al., 2020; Coco-Martin et al., 2021).

Currently, the MDR is established as a stricter regulation than the previous MDD. Additionally, the manufacturer will demonstrate a positive benefit–risk ratio for the product and compliance with the general safety and performance requirements. Therefore, it shall be shown from an experimental and clinical point of view that its tolerance is excellent and that its indications are fully justified by the corresponding scientific support (European Union Medical Device Regulation, 2023).

## 5 Conclusion

According to the chemical and biological evaluation of the Ala^®^sil infusion, signs of toxicity are evident and its clinical use was not justified by appropriate literature studies. All of this, together with a single clinical reference for this product, corroborate that the European Union’s medical device safety system was not working properly to assure safety. Fortunately, the current MDR 2017/745 applies stricter standards, including the medical personnel’s participation in evaluating the devices to have safer and more effective products. In addition, it efficiently promotes the CE marking as a clear sign of safety. In this way, the full implementation of the MDR will make it possible to avoid the commercialization of medical devices without high-quality preclinical and clinical information and precise clinical verification for their use, as has been demonstrated with the Ala^®^sil infusion.

## Data Availability

The original contributions presented in the study are included in the article/Supplementary Material, further inquiries can be directed to the corresponding author.
