# Supplementary material for: Ala®sil chemical characterization and toxicity evaluation: an example of the need for the Medical Device Regulation 2017/745
Source: Front Pharmacol. 2024 Jan 11;14:1310463. doi: 10.3389/fphar.2023.1310463 (PMC10811781; doi:10.3389/fphar.2023.1310463)
Supplement: Supplementary file 1 [file DataSheet1.docx]

Supplementary Material S1. Calibration curves for the LMWC.


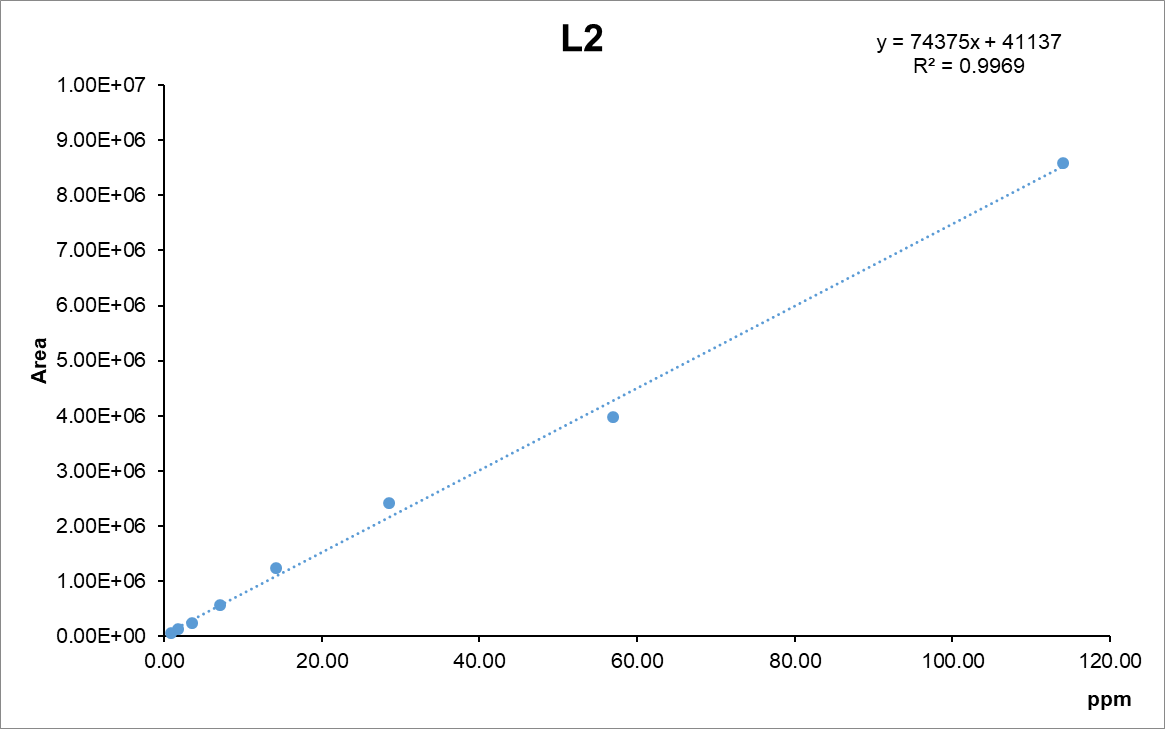


S1.1 Calibration curve for the L2 oligoxiloxane


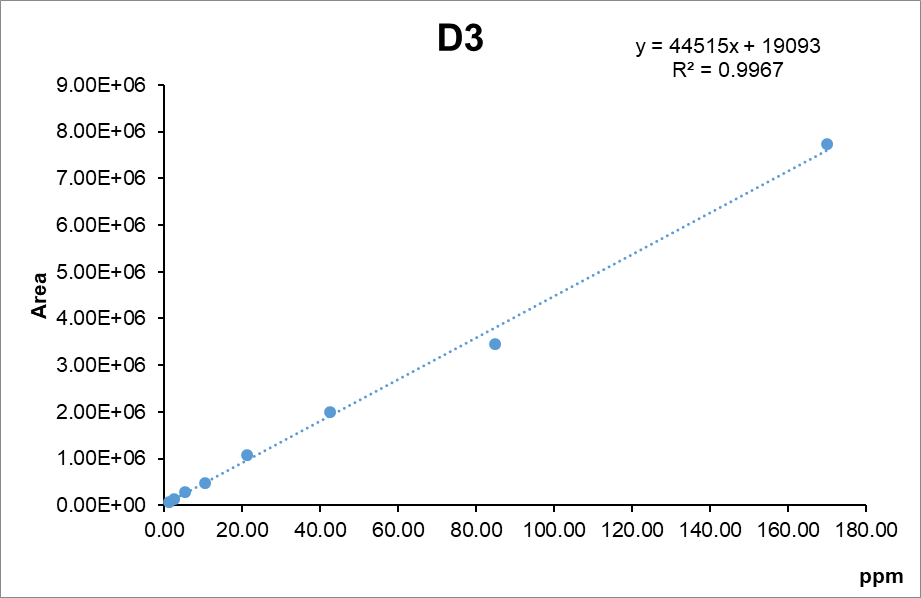


S1.2 Calibration curve for the D3 oligoxiloxane


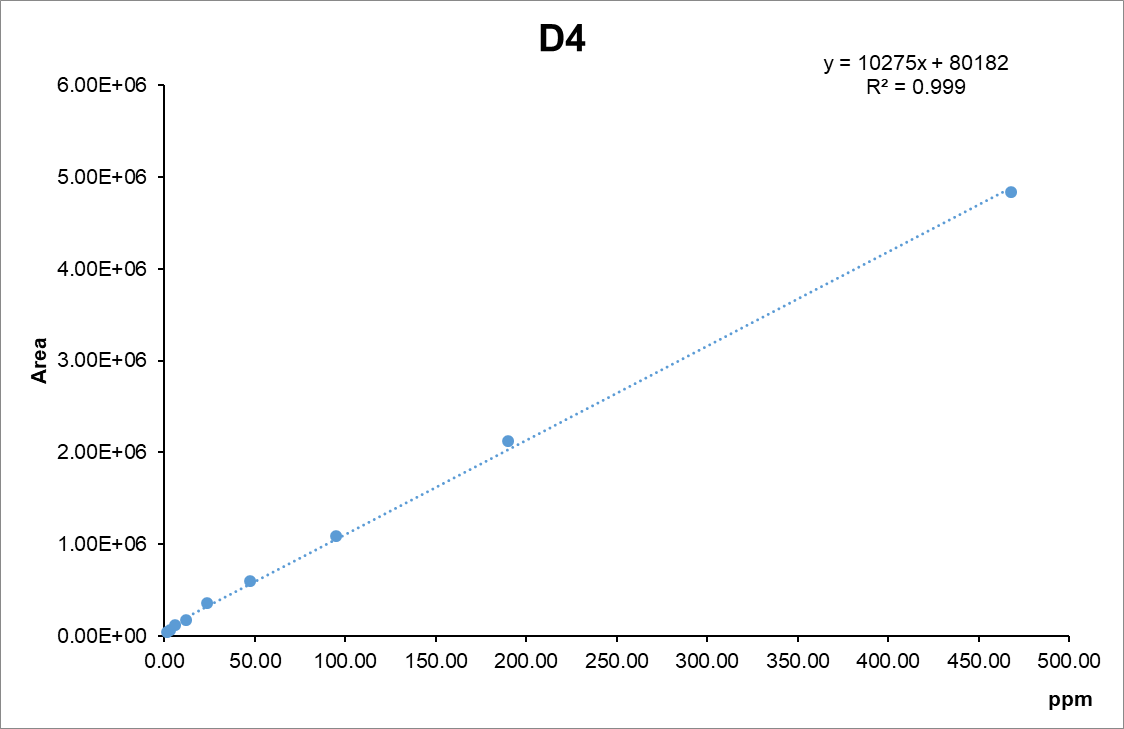


S1.3 Calibration curve for the D4 oligoxiloxane


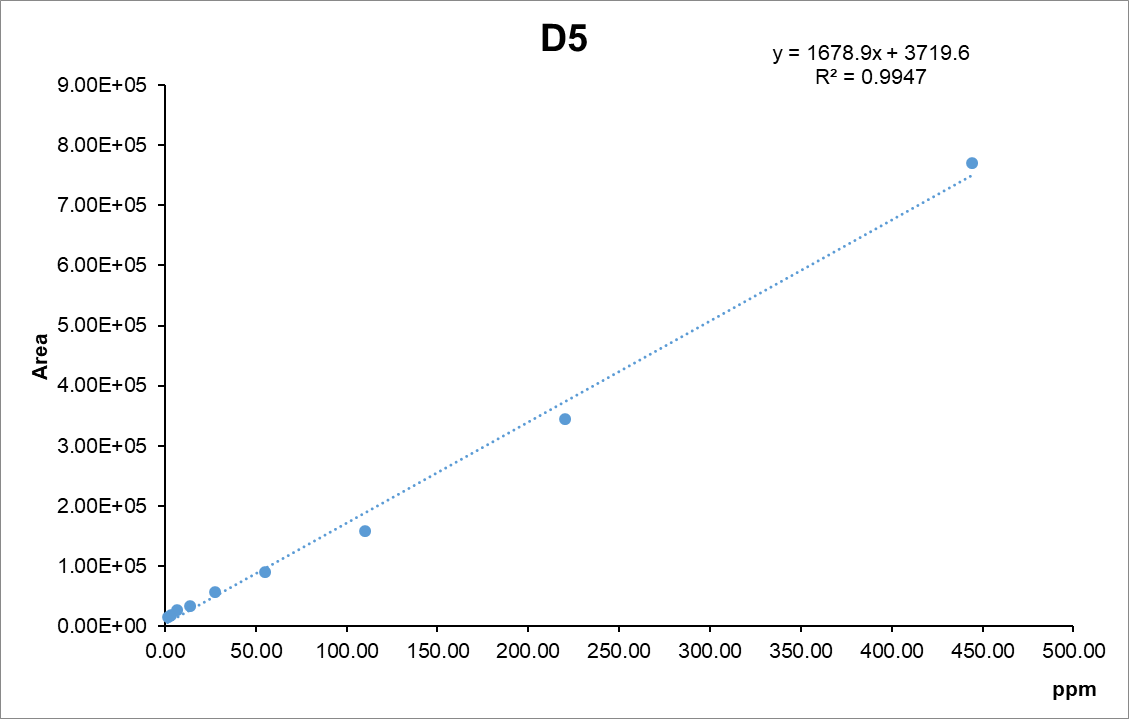


S1.4 Calibration curve for the D5 oligoxiloxane

Supplementary Material S2. MALDI results of low range m/z values.

Table S2.1 of the low mass peaks identified in MALDI-TOF.

| **m/z** | **S/N** | **Intens.** | **Area** |
| --- | --- | --- | --- |
| 127.2 | 79 | 12696 | 1089 |
| 137.8 | 113 | 20321 | 2136 |
| 138.2 | 494 | 88308 | 8873 |
| 147.2 | 56 | 10687 | 1070 |
| 150.2 | 74 | 14359 | 1250 |
| 192.1 | 34 | 7781 | 721 |
| 207.2 | 20 | 4858 | 1251 |
| 209.2 | 10 | 2472 | 543 |
| 211.1 | 18 | 4331 | 610 |
| 214.0 | 25 | 5917 | 619 |
| 215.0 | 50 | 11863 | 2210 |
| 217.2 | 39 | 9374 | 1009 |
| 221.1 | 70 | 17058 | 1970 |
| 223.1 | 14 | 3585 | 651 |
| 231.1 | 9 | 2228 | 283 |
| 245.1 | 20 | 5227 | 834 |
| 247.2 | 27 | 7068 | 1316 |
| 251.2 | 22 | 5698 | 606 |
| 259.2 | 24 | 6192 | 746 |
| 263.1 | 47 | 12250 | 1303 |
| 273.1 | 606 | 160078 | 22276 |
| 279.1 | 9 | 2522 | 456 |
| 281.1 | 20 | 5512 | 792 |
| 285.1 | 78 | 21034 | 2690 |
| 294.3 | 8 | 2392 | 272 |
| 295.1 | 9 | 2562 | 603 |
| 304.3 | 30 | 8273 | 948 |
| 313.1 | 40 | 10866 | 1298 |
| 317.1 | 9 | 2456 | 343 |
| 329.0 | 42 | 11385 | 1725 |
| 331.1 | 20 | 5321 | 629 |
| 332.4 | 8 | 2168 | 249 |
| 345.0 | 13 | 3429 | 697 |
| 345.2 | 12 | 3248 | 314 |
| 346.0 | 18 | 4851 | 500 |
| 348.2 | 43 | 11206 | 1468 |
| 349.2 | 19 | 5150 | 629 |
| 351.1 | 11 | 3032 | 442 |
| 359.1 | 9 | 2399 | 685 |
| 361.0 | 31 | 8095 | 1240 |
| 362.0 | 60 | 15392 | 2003 |
| 368.0 | 15 | 3883 | 463 |
| 369.0 | 25 | 6342 | 1062 |
| 375.1 | 27 | 6773 | 771 |
| 384.0 | 10 | 2496 | 262 |
| 391.0 | 9 | 2229 | 273 |
| 406.0 | 9 | 2215 | 252 |
| 425.1 | 14 | 2902 | 374 |
| 438.4 | 19 | 3688 | 515 |
| 454.4 | 42 | 7568 | 1084 |
| 522.5 | 16 | 2162 | 355 |
| 545.1 | 24 | 2823 | 405 |

Table S2.2 Comparison of the GC-MS characteristic m/z ions and ionization m/z values of the compounds identified in GC-MS in relation to the low mass ions in MALDI-TOF

| LMWC | [M] | [M+H]^+^ | [M+Na]^+^ | Correspondence with MALDI-TOF m/z low mass values (table S2.1) |
| --- | --- | --- | --- | --- |
| D3 | 222 | 223 | 245 | 223.1, 245.1 |
| L3 | 236 | 237 | 259 | 259.2 |
| D4 | 296 | 297 | 319 |  |
| L4 | 310 | 311 | 333 |  |
| D5 | 370 | 371 | 393 |  |
| L5 | 384 | 385 | 407 | 384.0 |
| D6 | 444 | 445 | 467 |  |
| L6 | 458 | 459 | 481 |  |
| D7 | 518 | 519 | 541 |  |
| L7 | 532 | 533 | 555 |  |
| D8 | 592 | 593 | 615 |  |
| L8 | 578 | 579 | 601 |  |


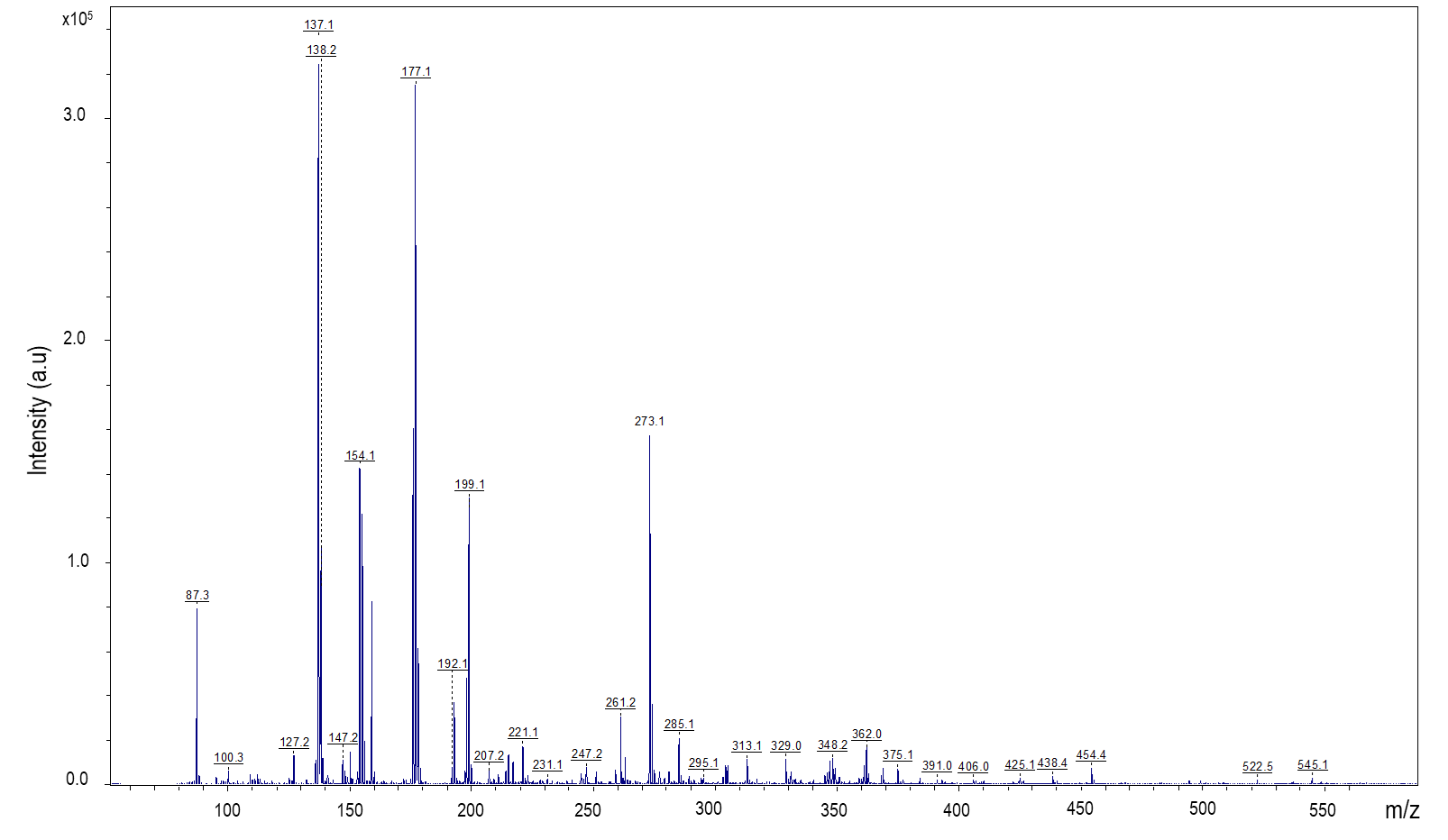


Figure S2.1: Spectra of the low range m/z from 0 to 600 m/z values of Ala®Sil sample.
